# Effects of antivirals on patients with COVID-19 breakthrough

**DOI:** 10.1186/s12879-023-08952-z

**Published:** 2024-01-02

**Authors:** Hong Tham Pham, Tuong-Anh Mai-Phan, Kim-Huong Truong-Nguyen, Minh-Hoang Tran

**Affiliations:** 1 Department of Pharmacy, Nguyen Tat Thanh University, 72820 HCMC, Vietnam; 2 Department of Surgical Gastroenterology, Nhan Dan Gia Dinh Hospital, 72316 HCMC, Vietnam; 3 Department of General Planning, Nhan Dan Gia Dinh Hospital, 72316 HCMC, Vietnam; 4 Department of Pharmacy, Nhan Dan Gia Dinh Hospital, 72316 HCMC, Vietnam; 5 NTT Hi-Tech Institute, Nguyen Tat Thanh University, 72820 HCMC, Vietnam

**Keywords:** COVID-19, Breakthrough Infections, Antiviral agents, Vietnam

## Abstract

**Background:**

Antivirals have been given widely for patients with COVID-19 breakthrough in Asian countries, creating a “black market” for unapproved and unprescribed medications. More evidence is needed to clarify the benefits of antivirals in these settings.

**Methods:**

We conducted a random-sampling retrospective cohort study at a general hospital in Vietnam. We recruited patients with mild-to-moderate COVID-19 breakthrough who were given either standard of care (SoC) alone or SoC + antiviral. Primary outcome was residual respiratory symptoms that lasted > 7 days. Secondary outcome was long COVID-19, diagnosed by specialized physicians. We used logistic regression to measure odds ratio (OR), in addition to a sensitivity and subgroup analyses to further explore the results.

**Results:**

A total of 142 patients (mean age 36.2 ± 9.8) were followed. We recorded residual symptoms in 27.9% and 20.3% of the SoC and SoC + antiviral group, while the figures for long COVID-19 were 11.8% and 8.1%, respectively. Antiviral use was not significantly associated with lower the risks of residual symptoms (OR = 0.51, 95% CI: 0.22–1.20, *p* = 0.12) or long COVID-19 (OR = 0.55, 95% CI: 0.16–1.90, *p* = 0.35). The sensitivity and subgroup analyses did not show any significant differences between the study groups (all *p* > 0.05).

**Conclusion:**

Antivirals were not associated with faster resolution of respiratory symptoms or lower risks of long COVID-19. Further studies should focus on different antivirals to confirm their effects on different sub-populations. Meanwhile, antivirals should only be used in very high-risk patients to avoid excessive costs and harms.

**Supplementary Information:**

The online version contains supplementary material available at 10.1186/s12879-023-08952-z.

## Background

The Coronavirus Disease 2019 (COVID-19) pandemic has spread to many regions worldwide, causing millions of deaths [1, 2]. Following the global vaccination rollouts, mortality rates have reduced significantly [3–6]. However, the number of breakthrough cases, defined as infection after ≥ 14 days of completing the primary series (± booster dose) [7], are still rising [8]. The waning of vaccine effectiveness over time and emergence of highly infectious variants are thought to be the primary causes [9, 10], which are, at least for now, unmodifiable risk factors. Therefore, breakthrough infection could affect many high-risk populations globally, especially in low- and middle-income countries (LMIC) where access to quality healthcare is often limited. The disease burdens might be more tremendous due to its long symptomatic period and sequelae.

Antivirals—while not being recommended for adults without risk of disease progression by the World Health Organization (WHO) [11], Infectious Diseases Society of America (IDSA) [12], or National Institute of Health (NIH) [13]—have been prescribed widely for patients with COVID-19 breakthrough in Asian countries like Vietnam. The justification for this was some believe antivirals could accelerate viral clearance, which could shorten the time to symptom-free status and prevent COVID-19 sequelae. Amid the COVID-19 outbreaks, this practice created a “black market” for unapproved and unprescribed antivirals.

No studies have reported the effects of antivirals on patients with COVID-19 breakthrough. Regardless of their benefits, uncontrolled use of antivirals could cause potential long-term harms (e.g., antiviral resistance, drug-induced neoplasm, etc.,) and unnecessary costs to the patients. To comprehensively address this issue, more evidence from LMIC settings is needed. Therefore, we conducted this study to investigate whether antivirals for COVID-19 treatments could help breakthrough patients improve faster or prevent associated sequelae.

## Methods

### Study design and participants

A retrospective cohort study was conducted at Nhan Dan Gia Dinh (NDGD) Hospital, a general tertiary hospital in Vietnam. Participant recruitment was taken by screening a sampling frame of patients under the management of NDGD Hospital from January 1, 2021, to January 31, 2022. We included patients who: (1) were ≥ 18 years old; (2) were fully vaccinated against COVID-19 before infection (received at least 2 doses, either homologous or heterologous, of the following vaccines: BNT162b2 (Pfizer/BioNTech), mRNA-1273 (Moderna), AZD1222 (AstraZeneca), or BBIBP-CorV (Sinopharm), at least 2 weeks before getting first COVID-19); (3) had a confirmative diagnosis of COVID-19 (positive to either real-time polymerase chain reaction test or rapid antigen test with typical symptom(s) of COVID-19); and (4) agreed to participate. Patients were excluded if they: (1) were pregnant or breastfeeding; (2) were severely or critically ill before treatment (based on the clinical spectrum proposed by the NIH [13]); (3) were moderately or severely immunocompromised (immunosuppressive medications, moderate or severe primary immunodeficiency, advanced or untreated human immunodeficiency virus infection, active cancer treatment, or white blood cell count < 4 × 10^9^/L); (4) were renally impaired (estimated glomerular filtration rate < 30 mL/minutes/1.73 m^2^); or (5) were hepatically impaired (Child–Pugh class B or C).

We followed the participants until March 31, 2022, or until they left the study. We reported this study in accordance with the Strengthening the Reporting of Observational Studies in Epidemiology (STROBE) Statement (Supplementary Checklist, available in the Supplementary File).

### Study groups

Two groups were investigated, of which patients were given: (1) standard of care (SoC, control group) or (2) standard of care plus antiviral (SoC + antiviral). In our study setting, SoC referred to treatment with appropriate medications (excluding antivirals) and supportive care that aligned with the guidelines of Vietnam’s Ministry of Health [14], WHO [11, 15], IDSA [12], and NIH [13]. Antivirals included remdesivir, molnupiravir, and favipiravir. Remdesivir was given by intravenous infusion to hospitalized patients, with 200 mg on the first day and 100 mg on the next 4 days. Molnupiravir was taken orally, with 800 mg twice daily for 5 days. Favipiravir was also an oral antiviral with dosage of 1,600 mg twice daily on the first day and 600 mg twice daily on the next days (duration of 5–7 days).

### Outcomes

The primary outcome was residual respiratory symptoms of COVID-19 breakthrough (including but not be limited to cough, dyspnea/shortness of breath/difficulty breathing, congestion, sore throat, loss of smell), measured in frequency. Based on our pilot data, the proposed timeframe cut-off to classify residual symptoms in COVID-19 breakthrough was 7 days. Thus, patients having respiratory symptoms after day 7 (from the day with first symptoms or diagnosis, whichever happened first) were counted towards the primary outcome. As these participants were under the management of NDGD Hospital, they were encouraged to self-report symptoms of COVID-19 every 1–2 days until resolution using MyCap platform [16]. For data collection, patients without self-reported records were contacted to retrieve the this outcome.

The secondary outcome was long COVID-19 [17–19], measured in frequency. This was diagnosed by specialized physicians in COVID-19 at NDGD Hospital using the guideline of the National Institute for Health and Care Excellence [19]. Following that, long COVID-19 includes ongoing symptomatic COVID-19 (“signs and symptoms of COVID-19 from 4 weeks up to 12 weeks”) and post-COVID-19 syndrome (“signs and symptoms that develop during or after an infection consistent with COVID‑19, continue for more than 12 weeks and are not explained by an alternative diagnosis”) [19]. We collected these data by screening patient health records for long COVID-19 diagnosis.

### Sample size

We calculated the sample size using the online website Power and Sample Size [20], with type I error rate (α) of 5%, power (1 - β) of 80%, and a sampling ratio of 1:1. Following the findings of Bergwerk et al., 31% of infected healthcare workers had residual symptoms 14 days after diagnosis [21]. Given that our study was conducted on the general population with a 7-day cut-off, we estimated the primary outcome could be found in at least 41% of the patients. For antivirals to be considered effective against COVID-19 breakthrough in low and middle-income countries like Vietnam, we expected a reduction of at least 50% in the primary outcome, resulting in a minimum sample size of 144 patients. Thus, we decided to recruit 150 patients.

### Covariates

Considering our study setting, the following factors were identified as potential confounders: gender (female/male), age (in years), weight (in kg), height (in cm), comorbidities, and concurrent medications. To avoid overadjustment bias, we excluded medications for comorbidities, keeping only those that were used for COVID-19 treatment.

### Statistical analysis

We removed observations that were missing or lost to follow-up from analysis. We presented demographic and baseline data as mean with standard deviation for continuous variables or as frequency with percentage for categorical variables. Incidence rates (using Poisson regression) and odds ratio (OR, using logistic regression) were given with 95% confidence intervals (95% CI). As there were 3 nationally approved antivirals for COVID-19 in Vietnam during this study timeframe (remdesivir, molnupiravir, and favipiravir), effect estimates might be biased by favipiravir due to its lack of evidence. To test the robustness of our findings, we conducted a sensitivity analysis by removing observations with favipiravir use. Since antivirals were primarily recommended for high-risk patients, we also wanted to explore these medications’ effects on both outcomes with a priori subgroup analysis. The subgroups were pre-specified based on the following variables: gender (male/female), age (< 65/≥ 65), comorbidities (yes/no), and corticosteroid use (yes/no). This subgroup analysis was considered exploratory to generate new hypotheses (if available), so we did not attempt to adjust for multiplicity. All statistical hypotheses were tested with a confidence level of 95%. We performed all analyses using R software (version 4.2.1, R Foundation for Statistical Computing, Vienna, Austria).

### Ethics approval

This study was approved by the Institutional Review Board of NDGD Hospital, Ho Chi Minh City, Vietnam, under approval number 85-2021/CN-HDDD. All recruited participants gave their informed consent.

## Results

### Patient characteristics

**Fig. 1:**
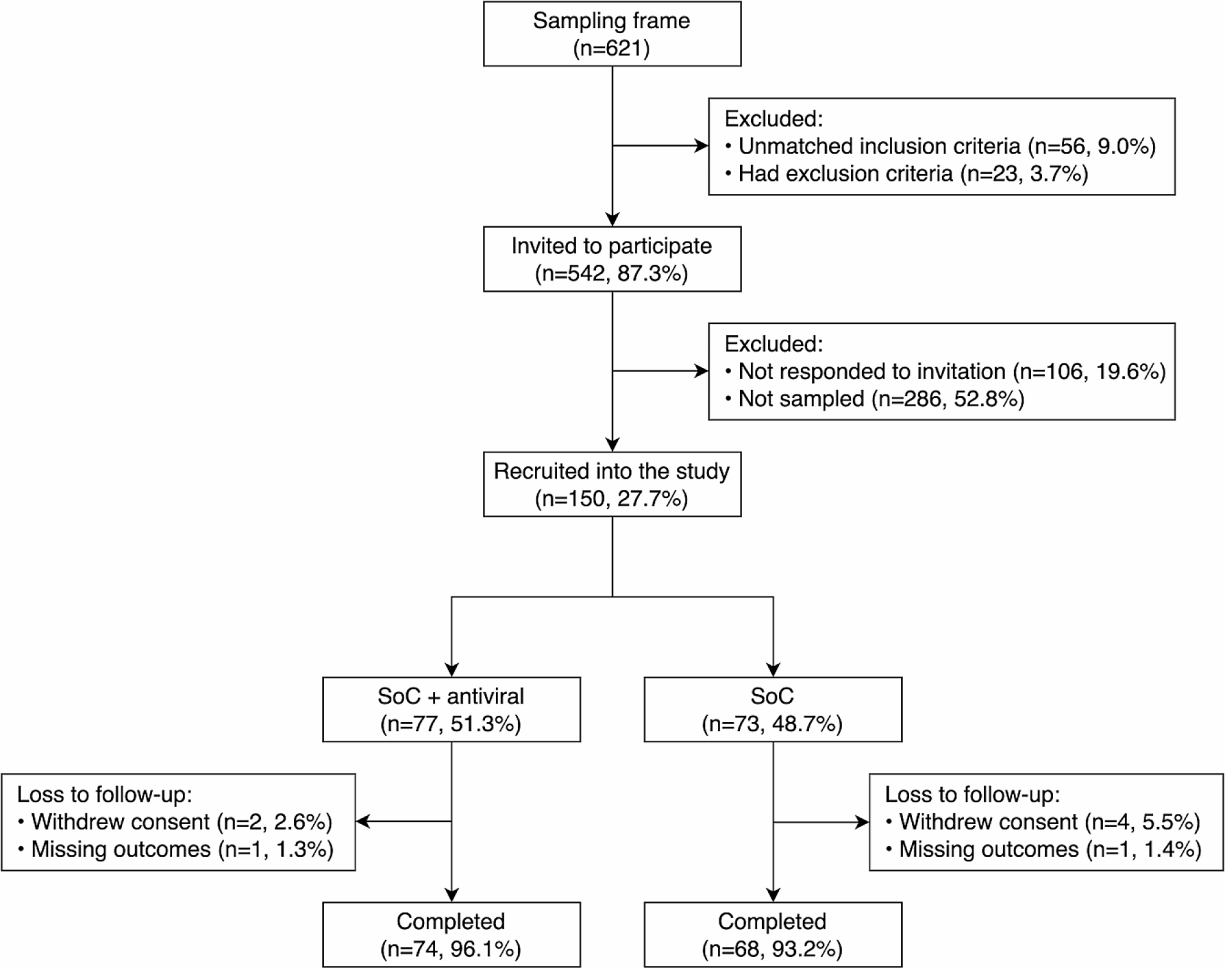
Flowchart of the participants *Abbreviation*: SoC: standard of care *Note*: Simple random sampling was used to generate the list of participants being recruited from those who agreed to participate. Among participants withdrawing consent, 2 moved away to other cities, and 4 traveled internationally. The other 2 participants could not be re-contacted after 3 consecutive follow-ups. Participants who were lost to follow-up were removed from the analyses

A total of 142 participants (average age 36.2 ± 9.8 years, 40.8% being male) completed the study (Fig. 1). The average body mass index was 23.3 ± 3.7 kg/m^2^, with 26.0% classified as obese (using the Asian adult cut-off [22]). Over one-fifth of participants had comorbidities, the majority being cardiovascular diseases (14 out of 32 cases). Most (93.7%) received homologous primary series, while only 12.7% had booster doses (mainly BNT162b2 vaccine). On average, symptoms of COVID-19 breakthrough or positive diagnosis appeared 54.8 ± 10.2 days after complete vaccination. Patients used various medications for symptom relief or severe outcome prevention, with antipyretics being the most commonly used (73.2%). Only 2.1% used anticoagulants. The primary choice of antivirals in the exposed group was molnupiravir (91.2%). Remdesivir was given to only 1 patient, while 5 others were prescribed favipiravir. Table 1 summarizes more information on baseline characteristics of the exposed and unexposed groups.

**Table 1:** Baseline characteristics

	SoC + antiviral (n = 74)	SoC (n = 68)	Overall (n = 142)
Male gender	34 (45.9)	24 (35.3)	58 (40.8)
Age (years)	37.3 ± 9.6	35.0 ± 9.9	36.2 ± 9.8
< 65	71 (95.9)	68 (100.0)	97.9
≥ 65	3 (4.1)	0 (0.0)	3 (2.1)
Weight (kg)	62.3 ± 13.1	58.6 ± 11.8	60.5 ± 12.6
Height (cm)	161.3 ± 7.7	160.0 ± 8.4	160.7 ± 8.0
Educational status			
Tertiary and above	69 (93.2)	59 (86.8)	128 (90.1)
Secondary and below	5 (6.8)	9 (13.2)	14 (9.1)
Comorbidities			
Endocrine diseases^1^	3 (4.1)	3 (4.4)	6 (4.2)
Cardiovascular diseases^2^	8 (10.8)	6 (8.8)	14 (9.9)
Respiratory diseases^3^	2 (2.7)	5 (7.4)	7 (4.9)
Gastrointestinal diseases^4^	3 (4.1)	0 (0.0)	3 (2.1)
Musculoskeletal disorders^5^	3 (4.1)	0 (0.0)	3 (2.1)
Cancers	0 (0.0)	3 (4.4)	3 (2.1)
First vaccine jab			
AZD1222^6^	70 (94.6)	59 (86.8)	129 (90.8)
mRNA-1273^7^	1 (1.4)	1 (1.5)	2 (1.4)
BNT162b2^8^	2 (2.7)	2 (2.9)	4 (2.8)
BBIBP-CorV^9^	1 (1.4)	6 (8.8)	7 (4.9)
Second vaccine jab			
AZD1222^6^	69 (93.2)	59 (86.8)	128 (90.1)
mRNA-1273^7^	1 (1.4)	1 (1.5)	2 (1.4)
BNT162b2^8^	3 (4.1)	5 (7.4)	8 (5.6)
BBIBP-CorV^9^	1 (1.4)	3 (4.4)	4 (2.8)
Concurrent medications			
Corticosteroids^10^	16 (21.6)	12 (17.6)	28 (19.7)
Anticoagulants^11^	2 (2.7)	1 (1.5)	3 (2.1)
Antipyretic agents^12^	62 (83.8)	42 (61.8)	104 (73.2)
Antibiotics^13^	19 (25.7)	9 (13.2)	28 (19.7)
Herbal medications^14^	10 (13.5)	15 (22.1)	25 (17.6)

*Abbreviation*: SoC: standard of care.

^1^Diabetes (type 2), hypothyroidism, hyperthyroidism.

^2^Hypertension, heart failure, coronary artery diseases, tachycardia, congenital heart diseases.

^3^Asthma, chronic obstructive pulmonary disease, allergic rhinitis, rhinosinusitis.

^4^Peptic ulcer, cirrhosis.

^5^Gout, osteoarthritis, osteoporosis.

^6^AstraZeneca vaccine.

^7^Moderna vaccine.

^8^Pfizer/BioNTech vaccine.

^9^Sinopharm vaccine.

^10^Prednisone, prednisolone, methylprednisolone, dexamethasone.

^11^Rivaroxaban, dabigatran.

^12^Paracetamol (acetaminophen), ibuprofen.

^13^Amoxicillin, amoxicillin-clavulanate, azithromycin, clarithromycin.

*Note*: All variables were reported with frequency (percentage), except for age, weight, and height, which were presented as mean ± standard deviation.

### Primary and secondary outcomes

**Fig. 2:**
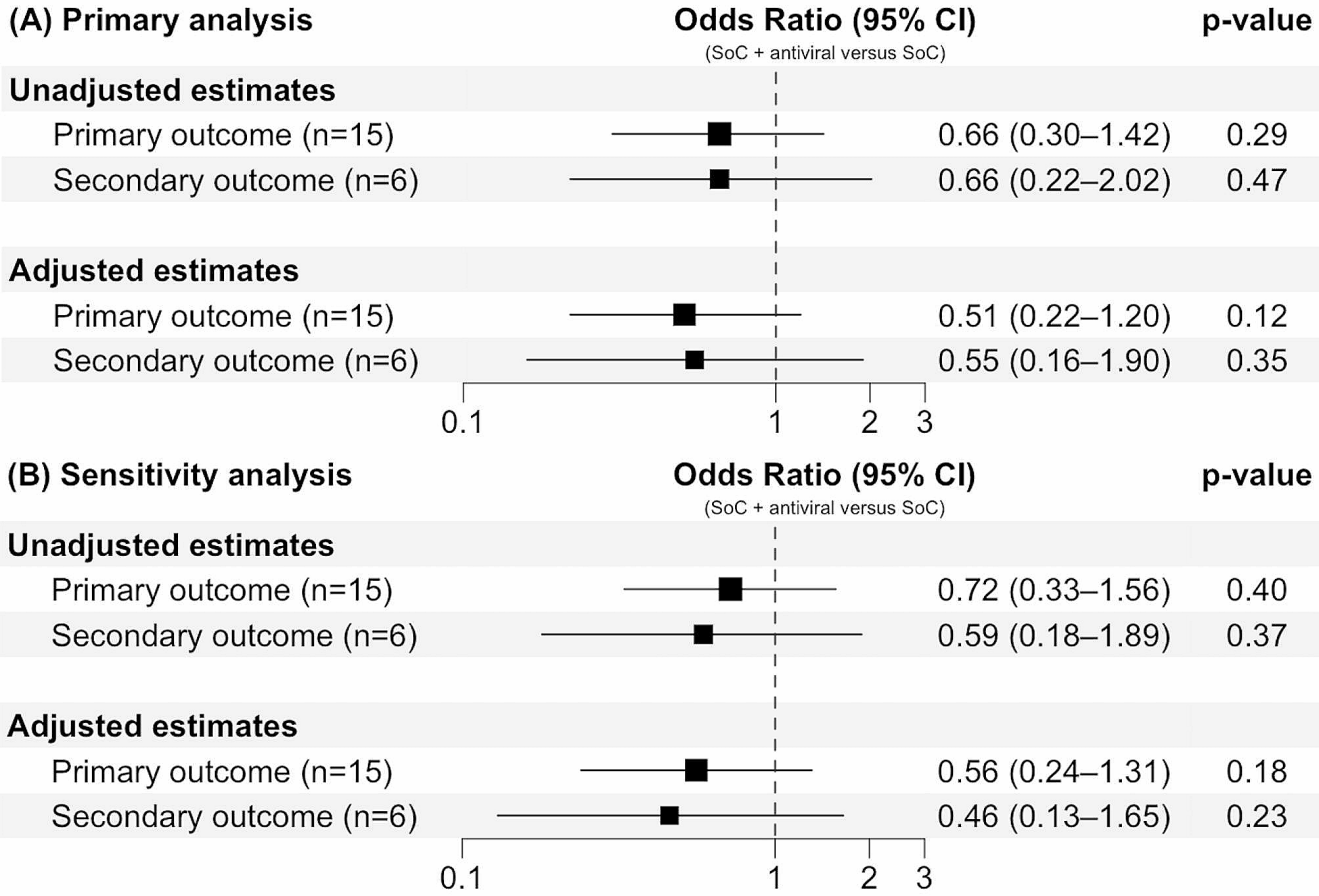
Effects of antivirals on the risks of residual respiratory symptoms (primary outcome) and long COVID-19 (secondary outcome) *Abbreviation*: CI: confidence interval; SoC: standard of care *Note*: Reference level of the exposure was SoC. Reference levels of the primary and secondary outcomes were symptom-free status within ≤ 7 days and no signs/symptoms of long COVID-19, respectively. Panel A: primary analysis. Panel B: sensitivity analysis. Adjusted estimates were controlled for gender, age, weight, height, comorbidities, and concurrent medications

There were 34 patients (23.9%) with the primary outcome, with an average duration of 11.1 ± 1.9 days experiencing respiratory symptoms. Asymptomatic infection was reported in 1 patient, while 2 required hospitalization after 7 days of home-based treatment. Antiviral-taking patients had lower incidence of residual respiratory symptoms than those who received SoC alone (20.3% versus 27.9%, Table 2), but the effect of antivirals were not statistically significant (OR = 0.51, 95% CI: 0.22–1.20, *p* = 0.12, Fig. 2, panel A).

**Table 2:** Incidences of residual respiratory symptoms (primary outcome) and long COVID-19 (secondary outcome)

	n	Primary outcome		Secondary outcome			
		**Event** **n (%)**		**Person-days** ^1^	**Event** **n (%)**	**Unadjusted rate** ^2^ **(95% CI)**	**Adjusted rate** ^2,3^ **(95% CI)**
SoC	68	19 (27.9)		2777	8 (11.8)	28.8 (12.4–56.8)	30.0 (14.6–61.6)
SoC + antiviral	74	15 (20.3)		2756	6 (8.1)	21.8 (8.0–47.4)	21.0 (9.1–48.3)
Favipiravir	5	0 (0.0)		69	1	144.9 (3.7–807.5)	300.0 (142.4–631.8)
Molnupiravir	68	14 (20.6)		2603	5	19.2 (6.2–44.8)	18.6 (1.7–198.2)
Remdesivir	1	1 (100.0)		84	0	–	–

*Abbreviation*: CI: confidence interval; SoC: standard of care.

^1^Outcome follow-up was available from January 1, 2021, to March 31, 2022.

^2^Rates are per 10,000 person-days.

^3^Poisson regression was used to adjust for gender, age, weight, height, vaccination status, comorbidities, and concurrent medications.

During a median of 21 days of follow-up (interquartile range 12.3–39.8), 14 diagnoses (9.9%) of long COVID-19 were given. Antiviral users had lower incidence rate of secondary outcome compared to SoC group (21.0 versus 31.0, per 10,000 person-days, Table 2). However, there was no evidence to support antivirals in preventing long COVID-19 among patients with breakthrough infection (OR = 0.55, 95% CI: 0.16–1.90, *p* = 0.35, Fig. 2, panel A).

### Sensitivity and subgroup analyses

**Fig. 3:**
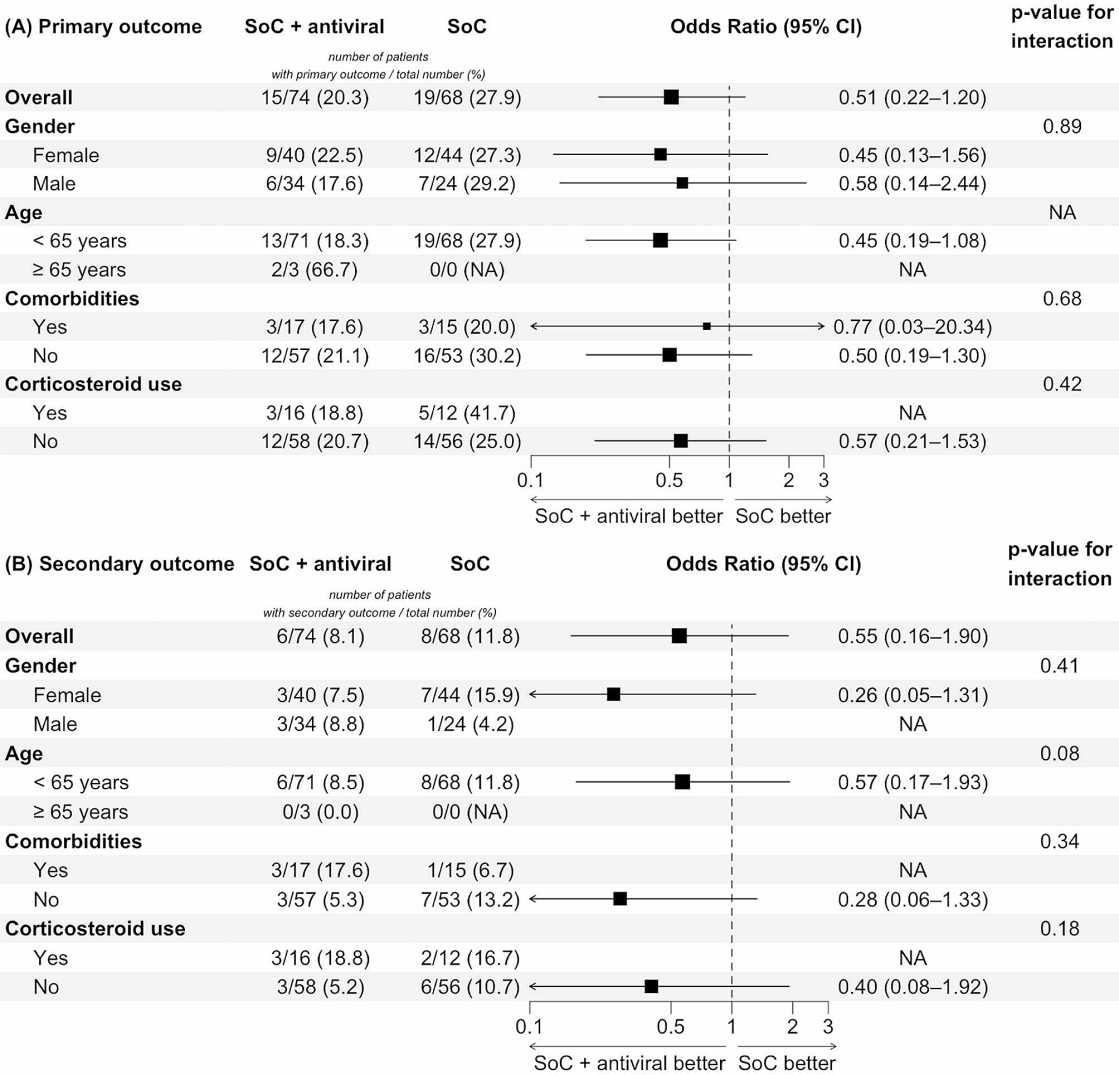
Effects of antivirals among subgroups on the risks of residual respiratory symptoms (primary outcome) and long COVID-19 (secondary outcome) *Abbreviation*: CI: confidence interval; NA: not available/not applicable; SoC: standard of care *Note*: NAs were due to no or too few events being observed. Reference levels of the primary and secondary outcomes were symptom-free status within ≤ 7 days and no signs/symptoms of long COVID-19, respectively. Panel A: primary outcome. Panel B: secondary outcome

Results of the sensitivity analysis were described in Fig. 2 (panel B). Excluding patients taking favipiravir did not lead to any significant differences in the outcomes between the exposed and unexposed groups. However, by looking at the changes in the effect estimates, we noticed that these 5 excluded observations were associated with a 9.8% increase in odds of residual symptoms and with a 16.4% decrease in odds of long COVID-19. This may suggest new hypotheses about the effects of favipiravir in patients with breakthrough infection. Following the subgroup analysis, all the stratified groups did not differ significantly (all ${\text{p}}_{\text{interaction}}$ > 0.05, Fig. 3). This implicated that there was little to no evidence favoring antivirals for high-risk patients with breakthrough infection, either in shortening symptom duration or preventing long COVID-19.

## Discussion

Overall, 20.3% of patients taking antiviral and 27.9% of those with standard of care alone experienced residual respiratory symptoms for more than 7 days, whereas the figures for long COVID-19 were 8.1% and 11.8%, respectively. Despite these lower incidences, antiviral use was not significantly associated with better outcomes. Types of antivirals also did not affect these associations notably.

While we could not confirm the benefit of antivirals in the time to symptom-free status, there is a likelihood that further studies with larger sample size may detect it, given our upper bound of the 95% CI: 0.22–1.20 (*p* = 0.12) is pretty closed to 1. Nevertheless, even with significant results, these benefits are perhaps not clinically important enough to inform the change in COVID-19 treatment guidelines, which only prioritize high-risk patients [11–13]. Meanwhile, fully vaccinated individuals tend to have lower risks of progression to severe outcomes [23]. Unless having critical risk factors, these patients may not resolve symptoms faster just by taking antivirals. Noteworthily, the excessive use of nirmatrelvir-ritonavir, an antiviral that has been approved for emergency use globally, could cause adverse effects by triggering rebound COVID-19 [24]. Therefore, antiviral prescription should not base solely on patient preference of faster symptom resolution.

Evidence of antivirals for long COVID-19 prevention is limited [25–27]. A recent study reported some potential benefits of nirmatrelvir-ritonavir, but no mechanisms under these effects were proposed [27]. In contrast, our molnupiravir-focused findings did not support antiviral use for this outcome, which is consistent with remdesivir’s effect in the Solidarity Finland trial [26]. It is unclear whether only nirmatrelvir-ritonavir can prevent long COVID-19 or its observed effects were due to some uncontrolled biases. Until we have enough evidence to clarify this hypothesis, antivirals should not be routinely recommended for patients at high risk for long COVID-19.

Our findings were also robust in terms of antiviral types. Favipiravir seemed to have no benefits on patients with COVID-19 breakthrough. However, as this study was not powered to detect favipiravir’s effects, it might be too soon to jump to the conclusion. Following our sensitivity analysis, we noticed that even if favipiravir could improve the outcomes significantly, its chance of being clinically important or cost-effective would be pretty small. Combined with the available evidence in unvaccinated patients with COVID-19 [28–30], we suggested against favipiravir for any sub-populations with COVID-19 until the emergence of better evidence.

To the best of our knowledge, this is one of the first study to investigate the effect of antivirals on patients with COVID-19 breakthrough. Evidence from our findings could aid in decision-making and form the foundations for later studies. However, some limitations still persist. First, our sample size was determined based on our expected clinical importance, which might not allow us to detect any statistical differences, if available. Second, incidence of long COVID-19 might have been underestimated as patients with troublesome symptoms tended to have medical examinations more frequently. Third, the study setting did not facilitate investigating the effects of nirmatrelvir-ritonavir, which could make the overall interpretation difficult and limit the applicability of the findings. Finally, we did not have sequencing data on the pathological variants, which may lower the specificity of our findings in other countries. Based on the predominance of delta and omicron during this study’s timeframe, our results could apply best to settings with the circulation of these variants.

## Conclusion

Although there were very limited benefits, antivirals were not associated with faster resolution of respiratory symptoms or lower risks of long COVID-19. Further studies should focus on different antivirals to confirm their effects on different sub-populations. Before strong recommendations are released, antivirals should only be used in very high-risk patients to avoid excessive costs and harms.

### Electronic supplementary material

Below is the link to the electronic supplementary material.

Supplementary Material 1

## Data Availability

The datasets used and/or analysed during the current study are available from the corresponding author on reasonable request.

## Abbreviations

95% CI: 95% confidence interval
COVID-19: Coronavirus Disease 2019
IDSA: Infectious Diseases Society of America
LMIC: Low- and middle-income countries
NDGD Hospital: Nhan Dan Gia Dinh Hospital
NIH: National Institute of Health
OR: Odds ratio
SoC: Standard of care
STROBE: Strengthening the Reporting of Observational Studies in Epidemiology
WHO: World Health Organization
